# Green synthesis of Fe₂O₃ from seedless lemon peel extract for anionic dye adsorption

**DOI:** 10.1186/s11671-026-04559-w

**Published:** 2026-04-13

**Authors:** Bich Ngoc Hoang, Chi Ngan Thi Vo, Thi Cam Quyen Ngo, Long Giang Bach

**Affiliations:** 1 Nguyen Tat Thanh University Center for Hi-Tech Development, Saigon Hi-Tech Park, Ho Chi Minh City, Vietnam; 2 Institute of Applied Technology and Sustainable Development, Nguyen Tat Thanh University, Ho Chi Minh City, Vietnam; 3 Faculty of Chemical Engineering and Food Technology, Nong Lam University, Ho Chi Minh City, 70000 Vietnam

**Keywords:** Adsorption, Lemon peel, Iron(III) oxide, Nanoparticles, Anion dyes

## Abstract

**Objectives:**

In this study, the Fe_2_O_3_ material was synthesized by green chemistry using seedless lemon peel extract. The structural characteristics of Fe_2_O_3_ were analyzed through SEM, FTIR, XRD and BET analytical methods. Factors affecting the adsorption of anionic dyes (Congo Red, Methyl Red, Methyl Orange) were also evaluated, such as temperature, pH, time, Fe_2_O_3_ dosage and dyes’ concentration.

**Data description:**

It was shown that the material has a heterogeneous cubic shape with iron and oxygen elements at the ratio of 43% and 12%. The crystal faces (120), (220), (400), (018), the material was identified as γ- Fe_2_O_3_ with a characteristic spinel structure with tetrahedral and octahedral shapes. The free functional groups on the surface are OH, Fe–O, with 635.58 cm^−1^ was attributed to the octahedral and tetrahedral sites of the Fe–O band. Temperature is the least influential factor and value was 30 °C. It can be seen that γ-Fe_2_O_3_ is the good adsorbent in the pH4–pH6 range, for 60 to 90 min, at dosage of 0.5 to 2 g/L and a dyes’ concentration of 200–300 mg/L. The maximum adsorption capacities of MO, CR, and MR were recorded 31.349 mg/g, 49.071 mg/g, and 119.705 mg/g, respectively. The adsorption mechanism has also been predicted and shown to depend on the type of dye with different interaction and adsorption capabilities. This helps the adsorption capacity of Fe_2_O_3_ material from lemon peel extract to be more diverse and applied more widely.

**Supplementary Information:**

The online version contains supplementary material available at 10.1186/s11671-026-04559-w.

## Introduction

The issue of protecting the earth has been receiving a lot of attention with different messages and aspects such as sustainable development, green energy, renewable energy, green products, etc. [1–5], 6, 7–9. In the study of Seerengaraj Vijayaram, an overview of nanomaterials synthesized from green solvents was given. In which, nanoparticles from gold, silver, iron oxide, selenium, copper were synthesized and used from many different extracts such as pummelo, Indian long pepper, onion, wild spinach, oregano, french tamarisk, butterfly tree, asian spider flower, thyme, water-hyacinth, lantana, sensitive plants, cultivated garlic, horseshoe geranium, fire lily, common grape, ceylon caper (Vijayaram et al. 2024). Nano materials have been studied a lot with different application possibilities such as dye treatment, photocatalysis, antibacterial, antioxidant, anti-cancer, and drug delivery [10, 11], 12].

Among them, Fe_2_O_3_ material synthesized by the green chemistry method was still quite limited in application in removing organic dyes. In addition, a larger amount of by-products from citrus trees was also released into the environment after processing. According to the report of author Divyani Panwar, the by-products after processing were peel (50–70%), pulp (60–65%), seeds (30–35%) and residue (less than 10%) [13, 14]. According to the Food and Agriculture Organization (FAO), the world citrus fruit supply in 2023 was reached 169.39 million tons, including oranges (41.23%), tangerines (31.03%), grapefruit (5.86%), lemons and limes (13.96%) and other citrus (7.9%). The application of citrus in food technology was developed 100 years ago [15]. Citrus fruits were not only used in food technology but also in many other fields [15]. Therefore, utilizing the by-products from citrus peels to synthesize Fe_2_O_3_ materials is one of the highlights of the research, and researchers have taken advantage of this issue to develop related studies. Fe_2_O_3_ was synthesized from some citrus peels that have been studied, such as lemon, tangerine, orange, and grapefruit peel extracts [16–18]. In the study of Tugce Aydogan, he used tangerine, orange, and grapefruit peels to synthesize Fe_2_O_3_ materials with the application of removing Congo Red with an efficiency of 36.5% [6]. In addition, Sithara's study also used orange peel extract to synthesize Fe_2_O_3_ materials and gave 97% photocatalytic capacity for methylene blue dye(Sithara et al. 2024b). The photocatalytic capacity also reached more than 97% with Fe_2_O_3_ materials using grapefruit peel extract in the study of Kamal Prasad Sapkota (Sapkota et al. 2025). It can be seen that the studies focused on grapefruit and orange peels and very little Fe_2_O_3_ used lemon peel extract [17–22]. Meanwhile, lemon peel contains many natural compounds such as flavonoids, limonoids, alkaloids, essential oils, pectin, and organic acids with ingredients such as limonene, 1-octanol, 3,7-dimethyl-1,6-octadien-3-ol (β-linalool), α-Terpineol, 3,7-dimethyl-6-octen-1-ol (cis-geraniol, β-citral, guariol (lemonol), (E)-3,7-dimethyl-2,6-octadienal [23]. In which compounds such as (E)-3,7-dimethyl-2,6-octadienal, cis-geraniol & β-linalool, α-Terpineol, and limonene contain aldehyde, alcohol, monoterpenoid alcohol, and monoterpene radicals that were easily oxidized when exposed to high temperatures, converted into acid radicals, and act as reducing agents. This contributes to the formation of Fe_2_O_3_ during synthesis from FeCl_3_. The use of green solvents helps the synthesis process avoid creating new pollutants. These were solvents containing compounds that act as natural acids for the synthesis of Fe_2_O_3_ materials. The Fe_2_O_3_ materials after the synthesis process were used to evaluate the ability to adsorb or remove organic dyes. In addition, factors affecting the adsorption process were also examined to determine the influence of adsorption conditions on the adsorption process of the material. Kinetic and isotherm models of adsorption were also used to predict the adsorption process or mechanism that may occur when the material adsorbs color molecules in water.

## Materials and methods

### Pretreatment and chemicals

The lemon peel is Citrus latifolia and was collected from a food processing company in Ben Luc, Long An province, Vietnam. After collecting, Lemon peel (LP) was washed to remove dirt and damaged parts. LP was dried at 50 °C for 48 h to remove water. Afterwards, LP was fine crushing into powder. Sodium hydroxide (NaOH), Iron (III) chloride hexahydrate (FeCl_3_.6H_2_O), and ethanol (≥ 95% purity) were produce from Xilong scientific Co., Ltd. Methyl Blue (MB), Brilliant Green (BG), Crystal Violet (CV), Methyl Orange (MO), Congo Red (CR), and Methyl Red (MR) were produce from Sigma-Aldrich Co, Switzerland.

### Preparation of extracted LP and synthesis Fe_2_O_3_

The LP extraction method and synthesis of Fe_2_O_3_ was based on Mohsan Bashir’s research and the previous research of the research group [22, 24]. 1 g of LP powder was soaked in 15 mL of 70% ethanol for 24 h, at room temperature. The LP extraction (Solution A) was then filtered to remove the residue and stored in sample tubes in a refrigerator at 10 °C ± 5 °C. Solution B was prepared by dissolving FeCl_3_.6H_2_O in 100 mL of deionized water and stirring at 70 °C for 60 min. 100 mL of solution A was added dropwise to solution B at 70 °C for 60 min. The mixture was further stirred for 30 min, and the pH was adjusted with NaOH solution to pH 7. The sample was centrifuged to collect the solid and washed several times with deionized water and ethanol to remove impurities and residual extract. After washing, the sample was dried at 70 °C for 24 h and ground to obtain Fe_2_O_3_ powder.

### Characterization

The Fe_2_O_3_ powder was evaluated for structural characteristics similar to those in the previous study including SEM, EDX, XRD, FTIR, specific surface area through specialized analytical equipment such as Hitachi S-4800 system, Japan (SEM, EDX), KBr powder coating with Nicolet 6700 Spectrometer (FTIR), Siemens D5000 diffractometer, Micro Active for TriStar II Plus 2.03 (High-throughput surface area and porosity analyzer). In addition, the adsorption process was evaluated on basic equipment and colour concentration was measured by a Metash UV-5100 spectrophotometer [22].

### Adsorption experiment

Adsorption evaluation experiments were performed based on the procedure performed in previous studies with some changes [22, 25, 26], 27]. 0.05 g of Fe_2_O_3_ powder and 100 mL of dyes solution (concentration of 25 mg/L, pH6) were added to 250 mL Erlenmeyer. Then, the sample was shaken in Incubator shaker (JEOTECH IST-4075R – Korean) at 30 °C for 2 h. The sample mixture was centrifuged 6000 rpm to recover the color solution after adsorption. Adsorption capacity (qe) and adsorption efficiency (H%) were calculated by applying formula (1) and (2). In which, the initial dye concentration (Co) and the equilibrium dye concentration (Cf) were determined by a UV–Vis spectrophotometer.

1 $${\mathrm{q}}_{\mathrm{e}}\left(\mathrm{mg}\cdot {g}^{-1}\right)=\frac{{(\mathrm{C}}_{0}-{\mathrm{C}}_{\mathrm{f}}) (mg\cdot {L}^{-1})}{\text{Dosage }(\mathrm{g}\cdot {L}^{-1})}$$

2 $$\text{H }\left(\mathrm{\%}\right)=\frac{\left({\mathrm{C}}_{0}-{\mathrm{C}}_{\mathrm{f}}\right) (mg\cdot {L}^{-1})}{{\mathrm{C}}_{0} (mg\cdot {L}^{-1})}\times 100$$

### The acids/bases surface and zeta potential measurement (pHpzc)

The pH point zero charge (pHpzc) was tested by the method reported in Bich's study [25, 28, 29], 30].

### Adsorption kinetics and isotherm model

The adsorption mechanism helps explain the interaction between Fe_2_O_3_ and dye through prediction from models. Kinetic and isothermal models were executed from the equations in the form of nonlinear models based on Quyen's research [27]. The model's coefficients with the adjusted R^2^ coefficient were calculated based on formulas and presented in Table 1.

**Table 1:** Equations of kinetic and isothermal models

Model name	Equation	References
*Adsorption isotherm*		
Langmuir	${q}_{e}=\frac{{q}_{m}{K}_{L}{C}_{e}}{1+{K}_{L}{C}_{e}}$ *(*${R}_{L}=\frac{1}{1+{K}_{L}{C}_{o}}$*)*	[27]
Freundlich	${q}_{e}={K}_{F}{C}_{e}^{1/n}$	[27]
Dubinin–Radushkevich (DR)	${q}_{e}={q}_{m}{e}^{-B{E}^{2}}$*(E* = $\frac{1}{\surd 2B}$*;* *ε* = *R.T.ln(1* + $\frac{1}{{C}_{e}})$*)*	[27]
Temkin	${q}_{e}={B}_{T}\mathit{ln}\left({K}_{T}{C}_{e}\right)$ *(*${B}_{T}=\frac{RT}{b})$	[27]
*Adsorption kinetics*		
Pseudo–first–order (PFO)	${q}_{t}={q}_{e}(1-{e}^{-{k}_{1}t})$	[27]
Pseudo–second–order (PSO)	${q}_{t}=\frac{{q}_{e}^{2}{k}_{2}t}{1+{k}_{2}t{q}_{e}}$	[27]
Bangham	${q}_{t}={k}_{B}{t}^{{\alpha }_{B}}$	[27]
Evlovich	${q}_{t}=\frac{1}{\beta }ln(1+\alpha \beta t)$	[27]

## Results and discussion

### Structural characteristics of Fe_2_O_3_

**Fig. 1:**
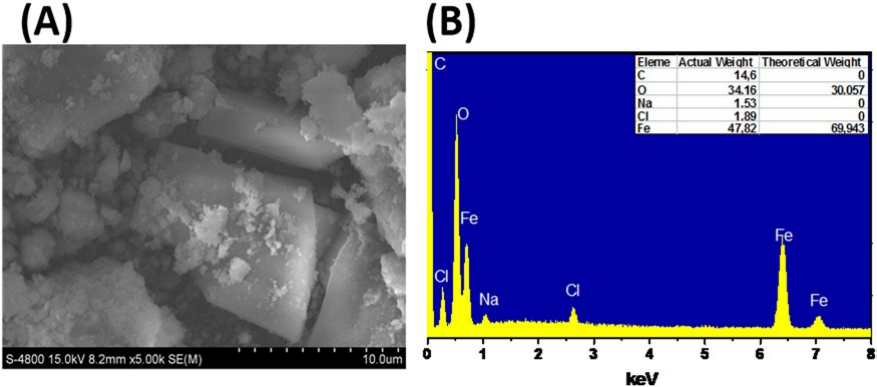
SEM images (**A**) and EDX (**B**) of Fe_2_O_3_ material from lemon peel extract at sizes of 10 µm (**A**)

The morphology of the iron oxide particles mediated by lemon peel extract was analyzed by scanning electron microscopy (SEM). The SEM results were shown in Fig. 1A with a 10μm scale bar. Fe_2_O_3_ material was irregular in size with a polyhedral shape and flat surface. Besides, the presence of small particles on the surface which were believed to be residual impurities of the extract after the synthesis process. This was also demonstrated similarly in the study of Virendra Kumar Yadava [31] and Ramesh Vinayagam [32]. EDX spectrum was determined the presence of elements in a sample by analysing the position and shape of the peaks in the spectrum, as shown in Fig. 4. The major species were labeled for the elements: Fe (Iron), Na (Sodium), Cl (Cl) and O (Oxygen). The results showed that Oxygen (O) accounts for 34.16%, Sodium (Na) accounts for 1.53%, Chlorine (Cl) accounts for 1.89%, (C) cacbon accounts for 14.6%, and Iron (Fe) accounts for 47,82% by mass. The results showed that the material sample could be a mixture of Fe_2_O_3_, FeOOH. Of which, Fe and O were the two major components present in the sample. Compared to the standard conditions of Fe_2_O_3_, the O ratio of 30.057% and the Fe ratio of 69.943% published in Mohsan Bashir's study showed similar results [24]. The low proportion of Fe and the presence of elements such as the Na, C and Cl can be considered as the formation of NaCl, and the remaining lemon extract (polyphenol and flavonoid) in the synthesis process. This confirms that the synthesized sample is Fe_2_O_3_. This was also demonstrated similarly in the study of Joy Sarkar [33].

**Fig. 2:**
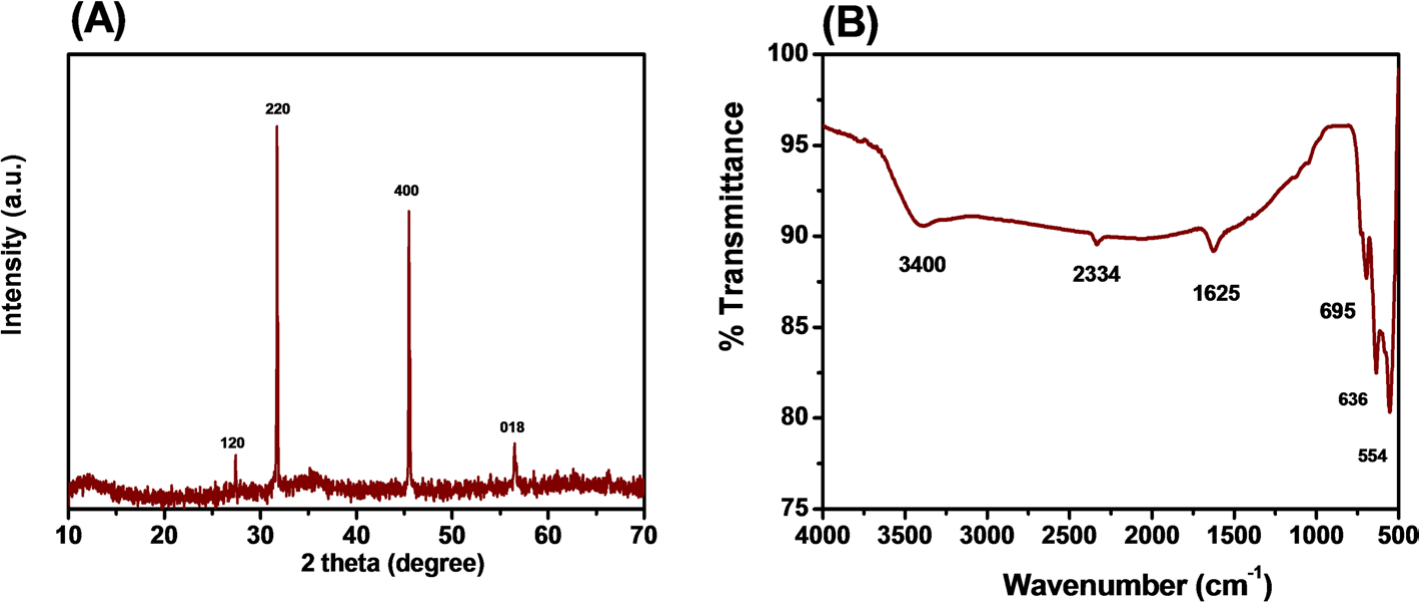
XRD (**A**) and FTIR (**B**) spectra of Fe_2_O_3_ from lemon peel extract

The crystal of Fe_2_O_3_ was evaluated and shown in Fig. 2A. The results show that the material had diffraction peaks at 2θ = 27°, 32°, 45°, 57° corresponding to the crystal planes (120), (220), (400), (018). In which, the (220), (400) crystal planes were characteristic of γ-Fe_2_O_3_, the (018), (120) crystal planes were the general crystal phase of Fe_2_O_3_ and bonding phase of the structure with the γ-FeOOH group partly for the structure of Fe_2_O_3_.This shows that the material had a γ- Fe_2_O_3_ crystal structure corresponding to the standard spectrum (JCPDS card number: 39-1346) characteristic of maghemite in the Shinde’s study [34]. The characteristic peaks were also studied similarly in previous studies [35–37]. Fourier transform infrared spectroscopy (FTIR) analysis was used to identify functional groups in a compound by integrating information about the specific vibrations of the bonds under investigation. The functional groups of the analyzed material were shown in Fig. 2B. The peak at 3425 cm⁻^1^ was considered to be the vibration of the -OH (hydroxyl) group, which was commonly found in hydrogen-bonded compounds such as water or organic compounds containing hydroxyl groups. The peak at 2334 cm⁻^1^ was chosen as the protocol of CO₂ or the stretching vibration of the C≡C or C≡N bond in compounds containing triple bonds. The peak at 1625 cm⁻^1^ was chosen as the vibration of the C=C bond in functional groups such as amides, esters or in aromatic rings. The peak at 695 cm^−1^ was related to the vibration of Fe–O in iron oxide [38]. The peaks 636, 554 cm⁻^1^ appearing in this spectral region represent the vibrations of the bonds between Fe atoms with atomic oxygen or chlorine (Fe–O, Fe–Cl). Among them, the peak 554 cm⁻^1^ was chosen as the protocol of the stretching length of the Fe–O mode, 636 cm⁻^1^ was assigned to the octahedral and tetrahedral sites of the Fe–O range [39]. The presence of hematite was also demonstrated similarly in the study of Kumar Brajesh [40]. In addition, the C≡C, C≡N and C=C groups are thought to be the aromatic ring, which belongs to the polyphenols (e.g., flavonoids and tannins) left over from the synthesis process, and in the study by Rabbia and Aleena [41, 42].

BET analysis showed in Table 2 that Fe_2_O_3_ had a surface area of ​​29.03 m^2^/g, a pore volume of 0.001 cm^3^/g, and a pore average size of 28.88 nm. The results showed that the surface area of ​​ Fe_2_O_3_ from lemon peel extract was quite low, and the value of pore volume was the lowest. This could be predicted because the Fe_2_O_3_ crystals did not overlap each other, thus limiting the formation of pores or voids between the crystals. Besides, the size was 28.8 nm, and compared with other studies, the crystals were large and flat surface. These results were consistent with what was shown in the SEM images.

**Table 2:** Surface area measurements of Fe_2_O_3_ from lemon peel extract

Sample	Solvent	Surface area (m^2^/g)	Pore volume (cm^3^/g)	Average size (nm)	References
γ-Fe_2_O_3_	lemon	29.03	0.001	28.88	This work
γ-Fe_2_O_3_	orange	41.488	0.362	34.90	[22]
Fe_2_O_3_	Tabebuia aurea	31.03	0.034	4.37	[43]
α-Fe_2_O_3_	green tea	22.5	0.034	15	[44]
Fe_2_O_3_	Bridelia retusa	75.19	0.119	7.92	[45]
α-Fe_2_O_3_	Pomegranate	31.52	–	5.54	[46]
α-Fe_2_O_3_	Moringa	58	–	20	[47]
α-Fe_2_O_3_	catechu	29.4	0.18	36.6	[48]

**Fig. 3:**
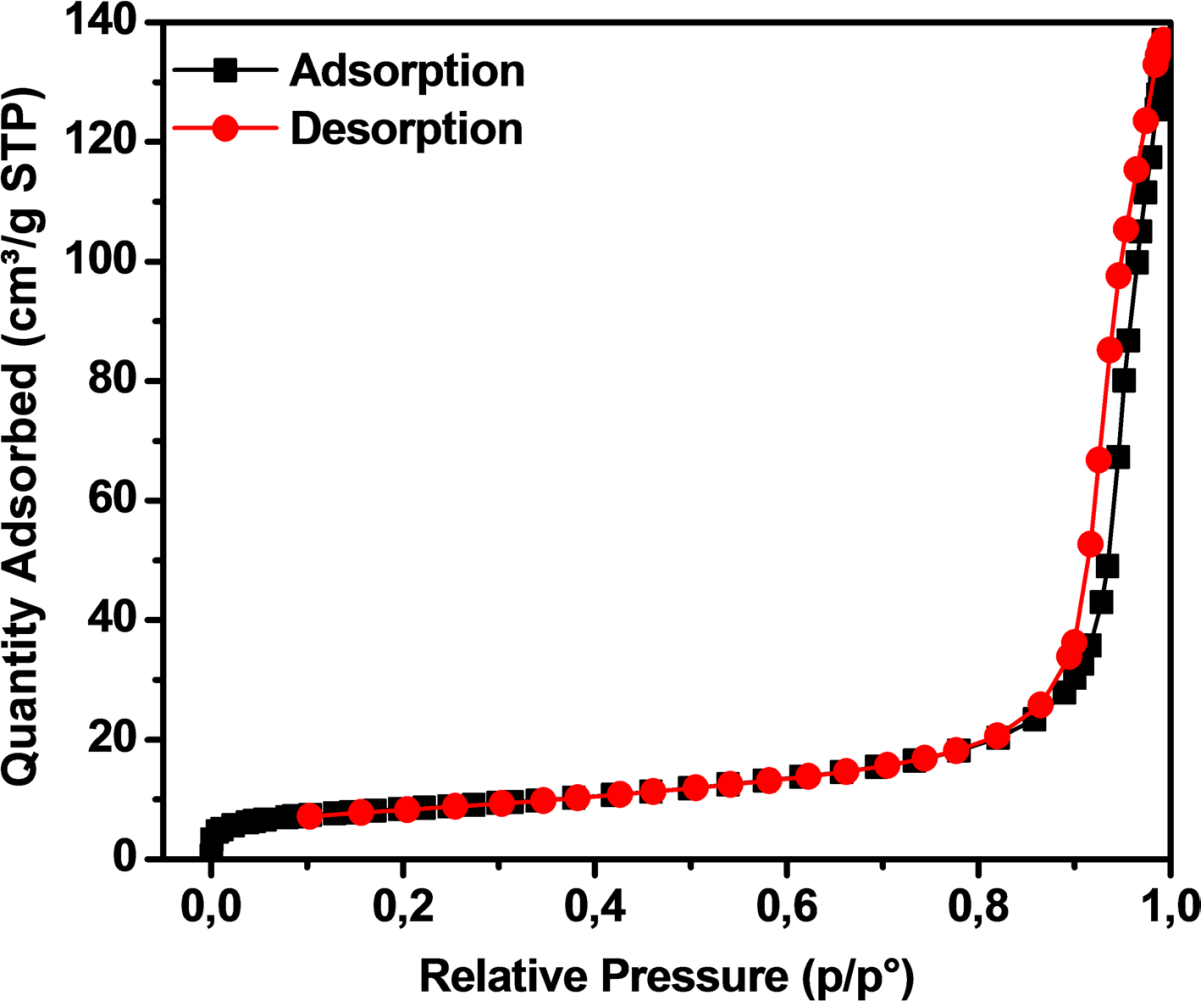
N_2_ adsorption and desorption isotherms of Fe_2_O_3_ from lemon peel extract

The N_2_ adsorption–desorption isotherm showing high adsorption amount was determined with pressure difference from 0 p/po to 0.01 p/po and 0.8 p/po to 1 p/po. The isotherm of the material at a very small pressure difference showed that the Fe_2_O_3_ had a large and flat surface. The adsorption isotherm characteristics of the material tended to follow type IV with hysteresis types H2, with the mechanism of gas molecules dispersing into the pores on the Fe_2_O_3_ surface with relatively high pressure (p/p° > 0.8, characteristic of capillary filling) and creating adsorption forces with the chemical bonds of the Fe_2_O_3_ surface. The adsorption process was described by constant adsorption species and parameters of the intensity of the adsorption process such as the maximum adsorption amount and the activation intensity of the adsorption process additive, according to the IUPAC adsorption isotherm classification standard (Fig. 3).

### Evaluation of selective adsorption

**Fig. 4:**
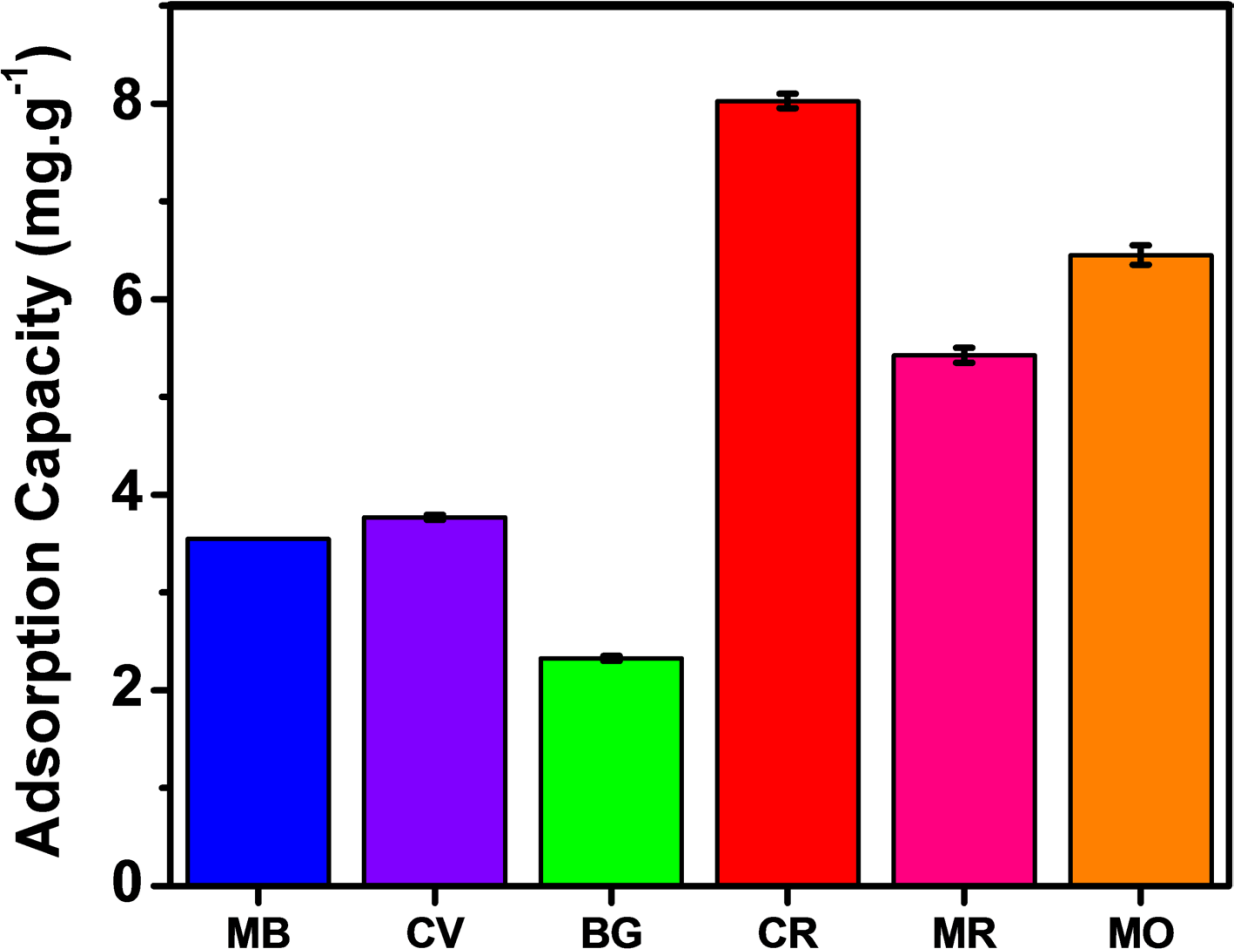
selective adsorption capacity of Fe_2_O_3_ from lemon peel extract

Evaluation of selective adsorption capacity was tested in six dyes with three cationic and three anionic dyes. Of which, Methylene Blue (MB), Crystal Violet (CV) and Brilliant Green (BG) were cationic dyes. Methyl Orange (MO), Congo red (CR) and methyl red (MR) were anionic dyes. The selection results in Fig. 4 show that the material had better adsorption capacity for anionic pigments than cationic dyes. Among anionic dyes, Fe_2_O_3_ was the adsorption capacity for CR of 8.025 mg/g, MR of 5.425 mg/g, and MO of 6.450 mg/g. To have an overview of the adsorption capacity of the materials for anion dyes, three dyes (including MO, CR, and MR) were selected to evaluate the adsorption capacity.

### Evaluation of influencing factors

**Fig. 5:**
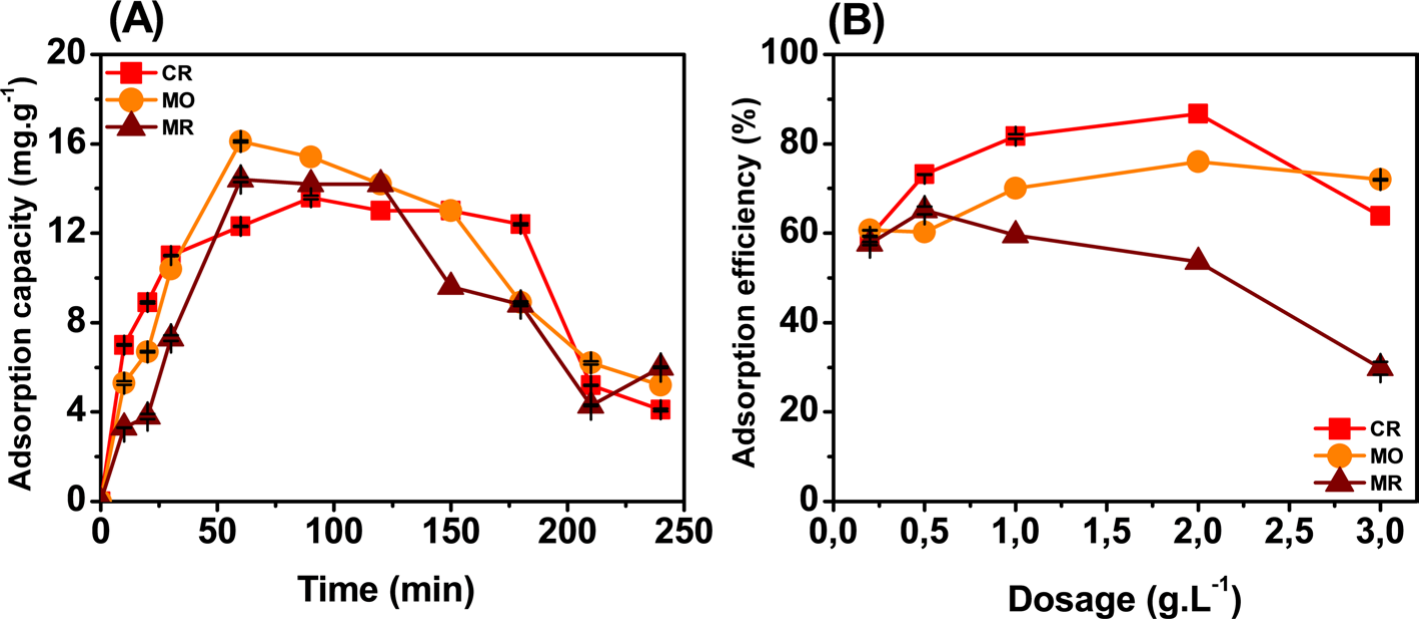
Effect of time (**A**) and Dosgae (**B**) on the adsorption capacity of Fe_2_O_3_ from lemon peel extract

In the adsorption process, the conditions such as time, pH, temperature, concentration and dosage were changed. The effect of time on the adsorption capacity for CR, MO and MR was evaluated in turn with the time range from 0 to 240 min as shown in Fig. 5A. The adsorption process occurred rapidly in the time period from 0 to 60 min through the rapid increase in adsorption capacity values ​​for all three dyes. For MO and MR, adsorption capacity was noted as unchanged and slightly reduced after 60 min. This indicates that adsorption has entered equilibrium for MO and MR from 60 to 90 min. Between 60 and 120 min, adsorption is still going on for CR dyes, adsorption capacity continues to increase and balance from 90 to 120 min. This suggests that the adsorption of Fe_2_O_3_ reaches a state of equilibrium that starts completely at 60–90 min The adsorption capacity starts to decrease at subsequent times. The adsorption capacity of Fe_2_O_3_ was recorded for MO (16.1 mg/g), CR (12.3 mg/g) and MR (14.4 mg/g) at the time point of starting the equilibrium process from 60 to 90 min and to evaluate for the following experiments.

The effect of Fe_2_O_3_ dosage on the adsorption capacity of CR, MO and MR was evaluated through Fig. 5B. The dosage was tested from 0.2 to 3 g/L and evaluated based on efficiency instead of adsorption capacity. This has been attributed to the fact that the adsorption calculation formula contains the dosage in the denominator which makes the evaluation no longer accurate, so the adsorption efficiency has been used to evaluate this factor. The results show that the adsorption capacity of MR increased from a dosage of 0.2 g/L to 0.5 g/L, corresponding to an increase in adsorption efficiency from about 60% to nearly 65%. From a dosage of 0.5 g/L to 3 g/L, the recorded efficiency decreased from 65 to 30%. This has been shown that at dosage of 0.5 g/L the highest MR adsorption level has been reached with an efficiency of 65% (30.6 mg/g). The adsorption efficiency of CR increased from 59 to 86% with dosage from 0.2 to 2 g/L, but this was decreased from 87.55 to 71.3% with dosage from 2 to 3 g/L. This has shown the best CR removal of Fe_2_O_3_ at a dosage of 2 g/L with an efficiency of 75% (8.15 mg/g). For MO, the initial adsorption efficiency was the same at the concentration of 0.2 and 0.5 g/L, but when the dosage increased to 2 g/L, the adsorption efficiency increased from 60 to 75%. When the dosage increased to 3 g/L, the adsorption efficiency was 72%. This has shown the best MO removal of Fe_2_O_3_ was about 70% (7.98 mg/g) and the best dosage value was 2 g/L. The results showed that the dosage value of 0.5 g/L was selected for MR, and 2 g/L was selected for CR and MO to evaluate the following factors.

**Fig. 6:**
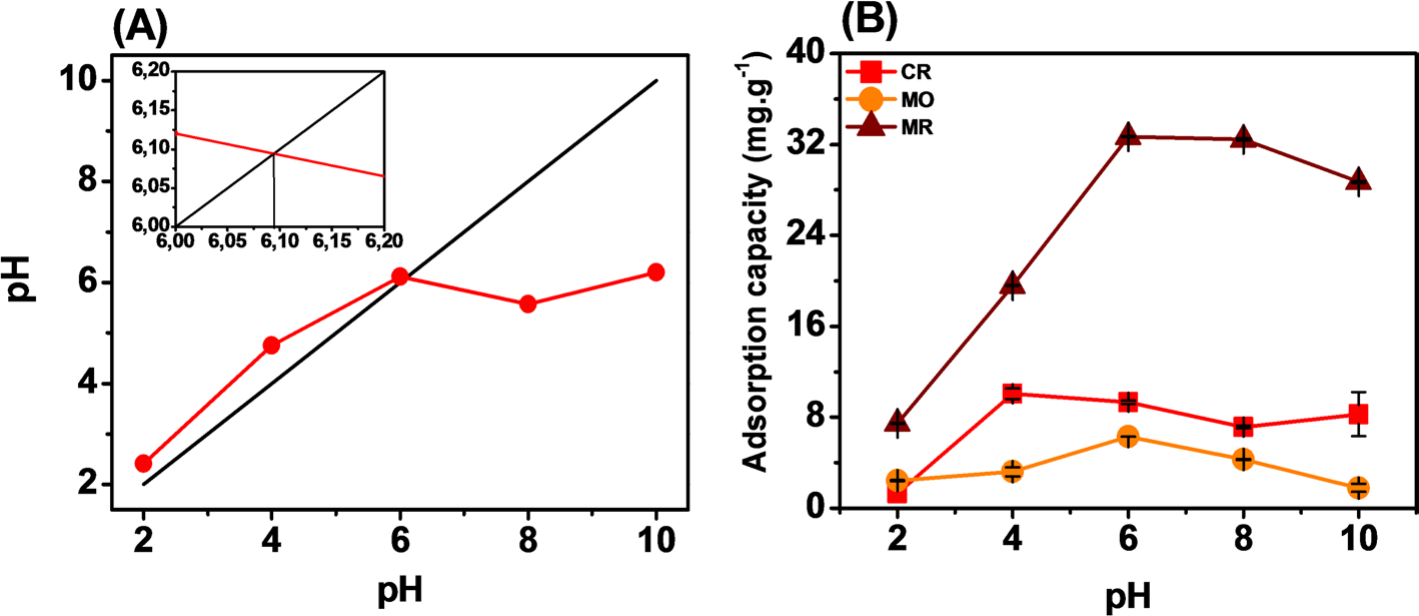
pHpzc value (**A**) and Effect of pH (**B**) on the adsorption capacity of Fe_2_O_3_ from lemon peel extract

The pH in solution was tested from pH 2 to pH 10. The pH values were determined using a pH meter and adjusted with NaOH and HCl solutions (0.01 M). The effect of the pH was tested and shown in Fig. 6. In which the pHpzc value of Fe was recorded at pH 6.1. The best adsorption capacities of Fe_2_O_3_ for the three dyes were recorded at pH 4 for CR and pH 6 for MR and MO, respectively. The pH value of the solution was smaller than the pHpzc value of Fe_2_O_3_ (positive charge due to protonated surface). This suggests that the adsorption of Fe to the three negative dyes is mediated by physical interactions (via Van der Waals forces, electrostatics, or pore-filling adsorption) and chemical interactions (hydrogen bonding, ion exchange, complexation, inner-sphere complex) [49, 50]. Therefore, pH4 và pH6 were chosen for MR, MO and CR to evaluate the subsequent adsorption coefficients.

**Fig. 7:**
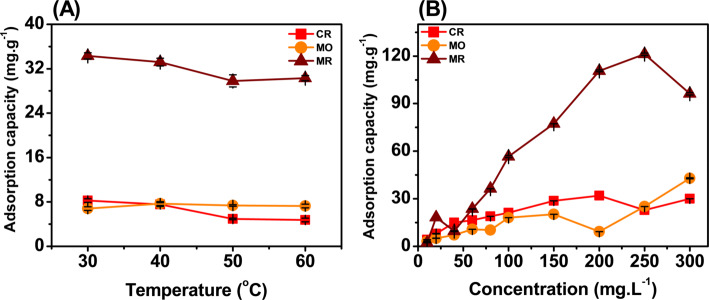
Effect of temperature (**A**) and Concentration (**B**) on the adsorption capacity of Fe_2_O_3_ from lemon peel extract

The effect of temperature and concentration on the adsorption capacity of Fe_2_O_3_ was tested and shown in Fig. 7. The adsorption capacity decreases with temperature from 30 to 60 °C. In MR, the adsorption capacity was recorded from 34 to 30 mg/g. Experiments performed on CR also gave similar results. Only MO showed a slight increase in adsorption capacity at 40 °C and a decrease when the temperature was raised to 50 oC, 60 °C. The best adsorption capacity was recorded at a temperature point 30 °C for CR (8.25 mg/g), MR (34.3 mg/g) and 40 °C for MO (7.7 mg/g). This shows that when the temperature increases, the disturbance of molecules inside the solution increases. This hinders the interaction between Fe_2_O_3_ and the dye molecules, causing the adsorption capacity to decrease. The rapid disturbance causes the interacting dye molecules to be dislodged, leading to the desorption process and the adsorption capacity was also recorded to decrease significantly [51, 52]. The results show that the temperature of 30 °C was chosen for MR and CR, and 40 °C for MO to evaluate the next factors.

The influence of the concentration of CR, MO and MR on the adsorption capacity of Fe_2_O_3_ was shown in Fig. 7B. The concentration was evaluated from 10 to 300 mg/L. For CR, the adsorption capacity increased from 10 mg/L (4.16 mg/g) to 200 mg/L (31.9 mg/g). This shows that the adsorption capacity increased insignificantly and the treatment efficiency was 29.6%. For MR, the adsorption capacity increased from 10 mg/L (2.3 mg/g) to 250 mg/L (121.2 mg/g). This shows that the adsorption process occurred rapidly with increasing dye concentration and the best adsorption capacity was recorded at 250 mg/L. This shows that MR dye was easily adsorbed by Fe_2_O_3_. For MO, the adsorption capacity increased from 10 mg/L (3.11 mg/g) to 300 mg/L (42.87 mg/g). Because the adsorption capacity still increased, MO was further evaluated at a concentration point of 350 mg/L, the adsorption capacity was recorded as 21.475 mg/g. This showed that MO dye was well adsorbed at a concentration of 300 mg/L. However, compared with the concentration of the dye before and after adsorption, the treatment efficiency of MO dye was about 21.26%. This showed that Fe_2_O_3_ did not treat MO and CR as well as other adsorbents such as activated carbon, composite film or Fe_2_O_3_ from orange peel [16, 18, 22, 53]. All factors have a certain influence on the adsorption process of Fe2O3 for the three types of pigments. In which, MR pigments are adsorbed better than CR and MO. This shows that there are many interactions between the material and MR, which makes the adsorption process easier than CR and MO pigments. In order to understand the interaction and process, adsorption isothermal and kinetics model experiments need to be performed. In summary, the best adsorption capacity of Fe_2_O_3_ was recorded with CR dye: time 90 min, temperature 30 ℃, pH4, concentration 2 g/L, concentration 200 mg/L. The best adsorption capacity of Fe_2_O_3_ was recorded with MR dye: time 60 min, temperature 30 ℃, pH6, concentration 0.5 g/L, concentration 250 mg/L. The best adsorption capacity of Fe_2_O_3_ was recorded with MO dye: time 60 min, temperature 40 ℃, pH6, concentration 2 g/L, concentration 300 mg/L. These were the best adsorption conditions chosen to predict the adsorption mechanism based on mathematical models.

### Evaluation of adsorption kinetics

**Fig. 8:**
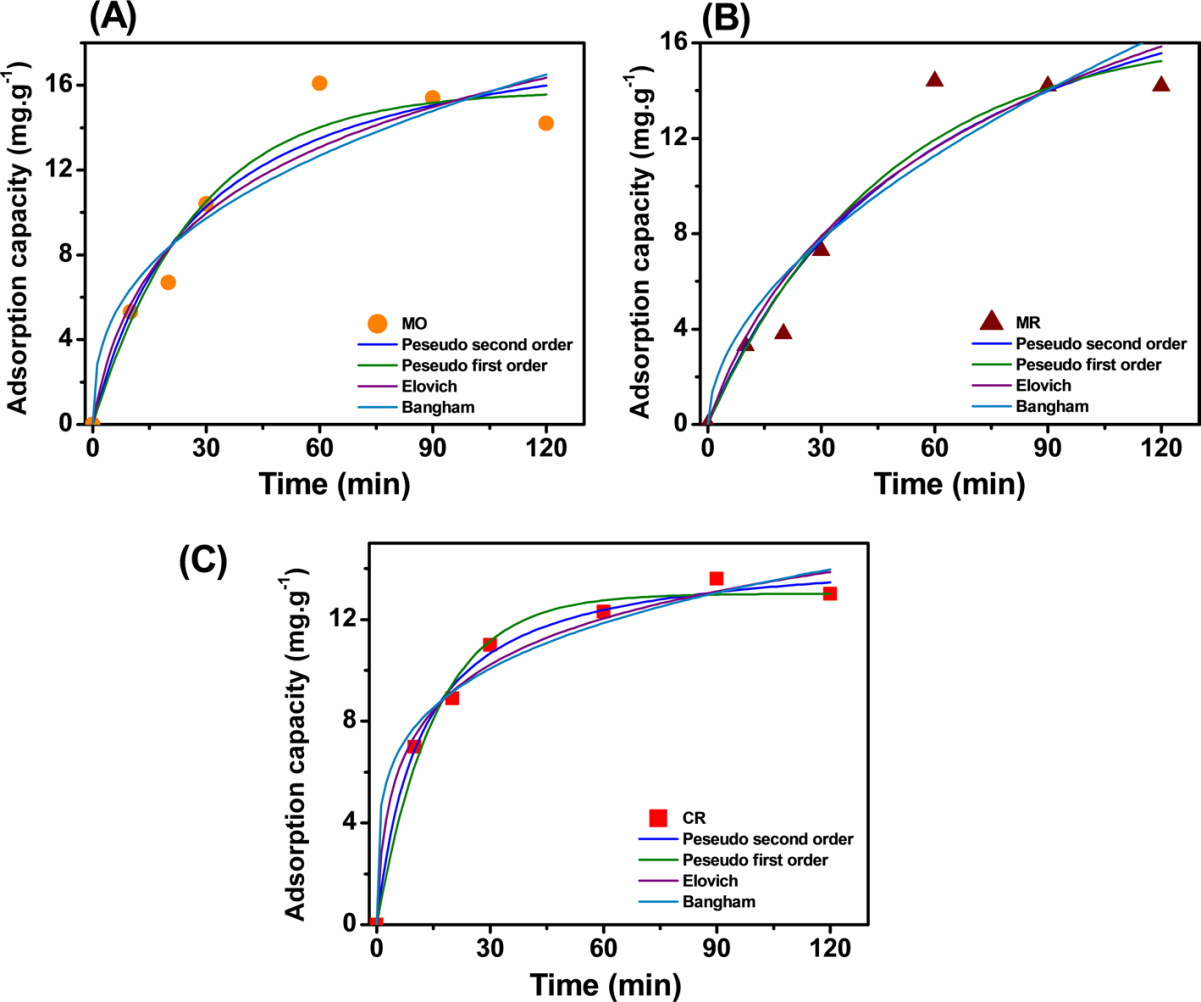
Adsorption kinetics of Fe_2_O_3_ from lemon peel extract with MO (**A**), MR (**B**), and CR (**C**) dyes

The adsorption kinetic model was used to predict the adsorption process of Fe_2_O_3_. The kinetic model was shown in Fig. 8, with the model parameters shown in Table 3. The experimental values ​​were evenly distributed on the graph corresponding to the kinetic lines. The model was fit assessed based on the model correlation coefficient (R^2^) recorded the highest value. For MO (Fig. 8A), the highest correlation coefficient was recorded as R^2^ = 0.952 corresponding to the PFO model. It could be seen that the MO adsorption process was mainly controlled by physical interactions. For MR (Fig. 8B), the highest correlation coefficient was recorded as R^2^ = 0.995 corresponding to the Elovich model. It could be seen that the MR adsorption process was mainly controlled by diffusion on a heterogeneous surface. In addition, the Bangham model also showed a certain agreement when R^2^ = 0.993 indicating that the adsorption process involves diffusion into the pores. It can be seen that the MR adsorption process was a diffusion process inside the pores at heterogeneous locations. For CR (Fig. 8C), the highest correlation coefficient was recorded as R^2^ = 0.993 corresponding to the PSO model. It can be seen that the CR adsorption process was mainly controlled by chemical interactions. In addition, the rate constant of the PSO model was very low. This showed that the initial adsorption process takes place by the attraction and interaction of functional groups on the Fe_2_O_3_ surface. It could be clearly seen that depending on the dye, Fe_2_O_3_ was adsorbed with different mechanisms.

**Table 3:** Kinetic parameters for the adsorption of Fe_2_O_3_ from lemon peel extract

Model	Parameters	CR	MR	MO
Bangham	k_B_ (mL/(g/L))	4.502	1.238	2.654
	α_B_	0.237	0.539	0.382
	R^2^	0.974	0.993	0.879
Elovich	β (g/mg)	0.376	0.137	0.200
	α (mg/g min)	4.036	0.474	1.052
	R^2^	0.983	0.995	0.907
PFO	q_e_ (mg/g)	13.017	16.512	15.752
	k_1_ (min^−1^)	0.065	0.021	0.037
	R^2^	0.986	0.939	0.952
PSO	q_e_ (mg/g)	14.742	23.629	19.621
	k_2_ (min^−1^)	0.006	0.001	0.002
	R^2^	0.993	0.926	0.930

### Evaluation of adsorption isotherm

**Fig. 9:**
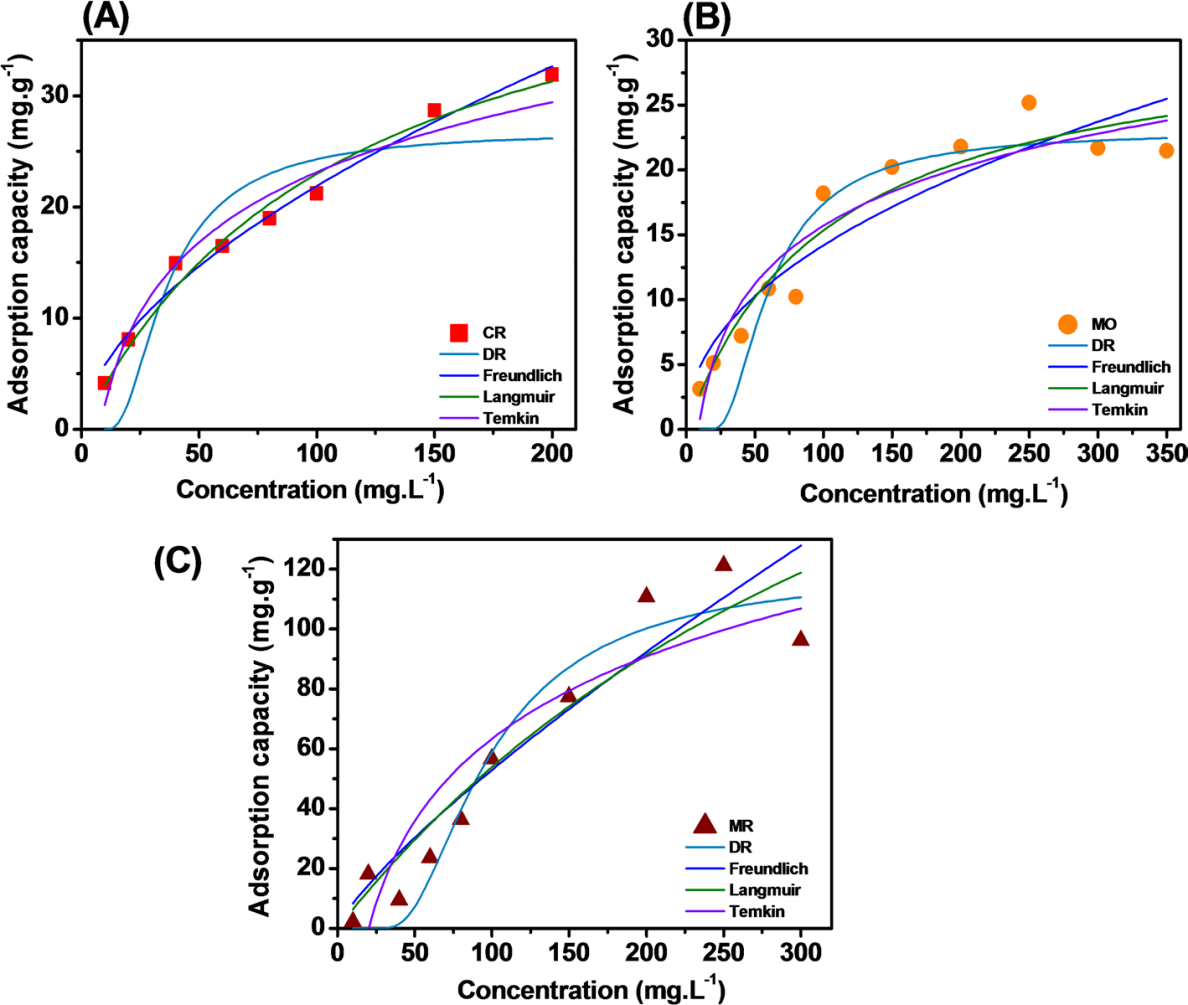
Adsorption isothermal of Fe_2_O_3_ from lemon peel extract with MO (**A**), MR (**B**), and CR (**C**) dyes

The adsorption isotherm model was used to determine the adsorption capacity and explain the mechanism of the association between the dye molecules into the adsorbent, the relative affinity of the dye molecules for the adsorbent. The Langmuir, Freundlich, Temkin and D–R adsorption isotherm models were presented in Fig. 9. The parameters obtained from the adsorption isotherm models of Fe_2_O_3_ with CR, MO and MR dyes were summarized in Table 4. The experimental values were evenly distributed on the graph corresponding to the isotherm lines. The model was fit assessed based on the model correlation coefficient (R^2^) recorded the highest value. For CR (Fig. 8A), the highest correlation coefficient was recorded as R^2^ = 0.982, corresponding to the Freundlich model. In addition, the maximum adsorption capacity recorded from the model was 49.071 mg/g. It can be seen that the CR adsorption process takes place on the monolayer surface. For MO (Fig. 9B), the highest correlation coefficient was recorded as R^2^ = 0.917, corresponding to the Langmuir model. In addition, the maximum adsorption capacity recorded from the model was 31.349 mg/g. It can be seen that the MO adsorption process takes place on the multilayer surface. For MR (Fig. 8C), the highest correlation coefficient was recorded as R^2^ = 0.931 and 0.929, corresponding to the Temkin and DR model. In addition, the maximum adsorption capacity recorded from the model was 119.705 mg/g. It can be seen that the MR adsorption process was carried out by the gaussian energy distribution on the heterogeneous surface. This shows that the adsorption process can be monolayer or multilayer, depending on the binding energy between Fe_2_O_3_ and MR dye molecules.

**Table 4:** Isotherm parameters for the adsorption of Fe_2_O_3_ from lemon peel extract

Model	Parameters	CR	MR	MO
Freundlich	K_F_ (mg/g)	1.536	1.308	1.648
	1/n	0.577	0.803	0.467
	R^2^	0.982	0.900	0.858
Langmuir	K_L_ (L/mg)	0.009	0.002	0.010
	Q_m_ (mg/g)	49.071	298.833	31.349
	R^2^	0.979	0.896	0.917
	R_L_			
Temkin	k_T_ (L/mg)	0.127	0.049	0.113
	B_T_	9.087	39.637	6.467
	R^2^	0.951	0.931	0.887
D-R	B (mol^2^/kJ^2^)	162.185	1166.343	457.162
	Q_m_ (mg/g)	26.824	119.705	22.962
	R^2^	0.753	0.929	0.856
	E (kJ/mol)			

Based on the Q_m_ parameters from the Langmuir model can see that the ability of materials to handle pigments is very different: MR (298.83 mg/g) > CR (49.07 mg/g) > MO (31.35 mg/g). Fe_2_O_3_ from lemon peel extract has an extremely impressive MR adsorption capacity. The figure of nearly 300 mg/g is a very high result for green synthetic materials, showing a special match between Fe_2_O_3_ structure with the Methyl Red molecule. Through the analysis of adsorption kinetics and isotherm models, the interaction ability of Fe_2_O_3_ materials with each type of dyes. For CR, the monolayer adsorption process with the interactions corresponds to the PSO and the Freundlich models. For MO, the multilayer adsorption process with physical interactions corresponds to the PFO and Langmuir models. For MR, the adsorption process was a diffusion process on the surface and inside the pores with the energy bonds corresponding to the Temkin, DR, Elovich and Bangham models. This can be explained by the unstable double bond of Oxygen in the structure when breaking the chain with organic compounds to create chemical interactions. In addition, the breaking of the oxygen chain causes Fe to have excess free charge, causing the formation of electrostatic interactions on the surface of the material. Because the material has an inert surface and a heterogeneous arrangement that creates many voids [54, 57].

## Conclusion

In this study, Fe_2_O_3_ material was synthesized by a green chemistry method using seedless lemon peel extract. Through SEM, FTIR, XRD and BET analysis methods, it was shown that the material has a heterogeneous cubic shape with iron and oxygen elements at the ratio of 43% and 12%. The crystal faces (120), (220), (400), (018), the material was identified as γ- Fe_2_O_3_ with a characteristic spinel structure with tetrahedral and octahedral shapes. The free functional groups on the surface were OH, Fe–O, with 635.58 cm⁻^1^ was attributed to the octahedral and tetrahedral sites of the Fe–O band. Factors affecting the adsorption process were also evaluated, in which temperature was the least influential factor, and the best temperature was 30–40 °C. In addition, pH, time, content and concentration were selected appropriately for the dye. It can be seen that Fe_2_O_3_ adsorbs well in the range of pH4-pH6, time from 60 to 90 min, content from 0.5 to 2 g/L and concentration from 200 to 300 mg/L. The maximum adsorption capacity of MO, CR, and MR was recorded 31.349 mg/g, 49.071 mg/g, and 119.705 mg/g, respectively. The adsorption mechanism has also been predicted and shown to depend on the type of dye with different interaction and adsorption capabilities. It can be seen that although they were all negative-based dyes, with different molecular structures, the Fe materials have different interactions on the surface. This was a highlight that needs further study to clearly evaluate the adsorption capacity of Fe_2_O_3_.

## Supplementary Information

Below is the link to the electronic supplementary material.

Supplementary Material 1.

## Data Availability

Data will be made available on request from the corresponding author.
