# Supplementary material for: Green synthesis of Fe₂O₃ from seedless lemon peel extract for anionic dye adsorption
Source: Discov Nano. 2026 Apr 13;21(1):117. doi: 10.1186/s11671-026-04559-w (PMC13076751; doi:10.1186/s11671-026-04559-w)
Supplement: Supplementary file 1 — Supplementary Material 1. [file 11671_2026_4559_MOESM1_ESM.docx]

**GRAPHICAL ABSTRACT**
